# How people come to recognise a problem and seek medical help for a person showing early signs of dementia: A systematic review and meta-ethnography

**DOI:** 10.1177/1471301215626889

**Published:** 2016-01-12

**Authors:** Lucy Perry-Young, Gareth Owen, Susan Kelly, Christabel Owens

**Affiliations:** School of Sociology and Social Policy, University of Nottingham, Nottingham, UK; University of Exeter Medical School, Exeter, UK; Egenis Centre for the Study of Life Sciences, University of Exeter, Exeter, UK; University of Exeter Medical School, Exeter, UK

**Keywords:** Alzheimer’s disease, dementia, help-seeking behaviour, illness behaviour, meta-ethnography

## Abstract

Evidence suggests that there is usually a long delay between noticing first signs of dementia and seeking medical help. We conducted a systematic review of what people experience and how they make decisions during this time, and used a meta-ethnographic approach to synthesise the findings. Screening and quality assessment resulted in nine studies eligible for inclusion. People with dementia mainly report experiencing memory lapses, while carers focus on more subtle changes in personality. People respond to these changes in one of three ways: 1) they discount them as normal; 2) they reserve judgement as to their cause and significance, or 3) they misattribute them. Pivotal events can finally trigger help seeking. Active reflection and seeking of further evidence may lead to earlier recognition of the possibility of dementia and the need to seek help; it also reduces the risk of a pivotal event. Public education should aim to improve recognition of more subtle signs and to encourage repeated evaluation and reflection.

## Introduction

Addressing the social and economic impact of dementia is one of the top UK and worldwide health priorities (Alzheimer's Society, 2009; Department of Health, 2009, 2012, 2013; Prince, Prina, & Guerchet, 2013). Although no cure currently exists for dementia, early diagnosis carries significant personal, social and economic benefits which have the potential to improve quality of life for patients and reduce carer burden (Banerjee & Wittenberg, 2009; Getsios, Blume, Ishak, Maclaine, & Hernández, 2012; Weimer & Sager, 2009).

In order for diagnosis to happen, a person, or someone close to them, must first identify a problem, associate that problem with dementia and make a decision to seek medical help. Although a general public population study indicated that people would seek help if they noticed memory problems in themselves or someone else (Dale, Hemmerich, Hill, Hougham, & Sachs, 2008), other studies, which are based on real as opposed to hypothetical situations, suggest a markedly different picture. For example, studies of real dementia trajectories reported averages of between 8 and 52 months from first signs to first medical consultation (Boise, Morgan, Kaye, & Camicioli, 1999; Bond et al., 2005; Chrisp, Thomas, Goddard, & Owens, 2011; Clark et al., 2005; Drebing et al., 2004; Speechly, Bridges-Webb, & Passmore, 2008; Wackerbarth & Johnson, 2002; Watari & Gatz, 2004). It is likely that these numbers are an underestimate of the actual length of delay because participants in all of the studies *had* eventually sought a diagnosis.

Several possible explanations have been offered for this delay. Firstly, the symptoms of dementia can be difficult to recognise. In a survey of older people meeting criteria for dementia, Eustace et al. (2007) found that 29% of primary informants failed to recognise changes in their loved one’s memory. The attribution of memory problems to normal aging is also widely reported (Bond et al., 2005; Braun, Takamura, & Mougeot, 1996; Clare, 2003; Clark et al., 2005). Other recognised reasons for delay are: difficulty discussing symptoms with the person with dementia (Clark et al., 2005); difficulty accepting dementia (Boise et al., 1999; Clark et al., 2005); the person with dementia being angry or refusing to see a doctor (Clark et al., 2005); desire to protect the autonomy of the person with dementia (Boise et al., 1999); family disagreements about the problem and the course of action (Boise et al., 1999); and difficulty talking with the doctor about mental health issues (Boise et al., 1999).

These reasons for delay in help seeking are echoed by the findings of studies into help seeking for mental health. This literature suggests that the most common reasons for delay in help seeking are: stigma and embarrassment (Gulliver, Griffiths, & Christensen, 2010; Meltzer et al., 2000), difficulty recognising symptoms (Clausen & Yarrow, 1955; Gulliver et al., 2010), self-reliance and/or preference for lay help (Oliver, Pearson, Coe, & Gunnell, 2005; Rickwood, Deane, Wilson, & Ciarrochi, 2005), lack of ability to express emotions (Gulliver et al., 2010; Meltzer et al., 2000; Rickwood et al., 2005), the view that emotional problems are not a legitimate illness (Pill, Prior, & Wood, 2001; Prior, Wood, Lewis, & Pill, 2003), doubts about the usefulness of seeking help (Meltzer et al., 2000; Pill et al., 2001) and the fear of consequences of seeking help (Dew et al., 2007; Owens et al., 2011).

The wider help-seeking literature has shown that people respond to the signs of illness in diverse and often unexpected ways. While it might seem obvious that a person seeks help when he/she is ‘sick’, what constitutes sickness and what is considered to be appropriate action in response to sickness varies widely between individuals and groups (Zola, 1973). Zola (1973) challenges three major and common assumptions about the reasons individuals seek a medical consultation. The first assumption is that most people at most times in their lives are asymptomatic. We know however from studies in which family health diaries were kept (e.g. Robinson (1971)), that ‘we may to some degree be sick every day of our lives’ (Zola, 1973, p. 678). The second assumption is that the more serious or frequent the symptoms, the more likely people are to seek help. Zola challenges this assumption by highlighting the evidence that, for serious physical or mental disorder, there is often at least one person out of treatment for every person in treatment. The third assumption is that a rational individual experiencing symptoms would, after a time, seek a medical consultation. Here Zola highlights the studies of delay in seeking help for cancer.

Others have demonstrated that most people rely heavily on personal social networks as a first ‘port of call’ in seeking help, noting that many illness episodes are recognised and managed within these networks without professional help (Cornford & Cornford, 1999; Freidson, 1970; Kleinman, 1980; Scambler, Scambler, & Craig, 1981). Since these early studies, several ‘dynamic’ models, which acknowledge the role of social networks, have been proposed to describe the ways in which individuals make decisions about whether or not to seek professional help (Biddle, Donovan, Sharp, & Gunnell, 2007; Dingwall, 1976; Leventhal, Nerenz, & Steele, 1984; Pescosolido, 1991, 2006; Saint Arnault, 2009; Wyke, Adamson, Dixon, & Hunt, 2013). These dynamic models highlight that changes are interpreted in light of knowledge gained through interactions over time (Wyke et al., 2013), that changes are observed, attributions made about their cause, and their social significance is determined (Saint Arnault, 2009), and that the threshold for defining the need for help may be movable (Biddle et al., 2007). Despite the wealth of insight these models provide, they do not fully account for situations in which family and social network members play a more active role in recognising that something is wrong and seeking help on behalf of another person. These situations may be particularly common in some mental health conditions, including dementia, where illness is characterised by a lack of awareness.

Further exploration is needed to understand how people come to recognise a problem and seek professional help for individuals showing early signs of dementia. There has been no attempt to systematically review and synthesise the qualitative literature on the help-seeking behaviours of people with dementia and members of their family and social networks.

### Aim and research question

The aim of this review was to systematically search, critically appraise and present a synthesis of the literature on the social dynamics of help seeking for dementia. The specific research question was: How do people experience and interpret early signs of dementia and make decisions about whether and when to seek medical help?

## Methods

We used systematic reviewing techniques to search for, and appraise the quality of all qualitative studies that were relevant to the research question. After data were extracted, the findings of these papers were synthesised using a meta-ethnographic method (Noblit & Hare, 1988).

### Search strategy

The search strategy (Appendix) was built up using the Ovid platform and was amended where necessary to work in other databases. The following databases were searched from their inception up to May 2015: MEDLINE, PsycINFO, Embase, CINAHL, HMIC, British Nursing Index, Social Care Online and ASSIA. This produced 3650 hits. After removal of duplicates, two reviewers (LP-Y and GO) independently screened titles and abstracts to assess eligibility according to the inclusion and exclusion criteria. The reviewers met to compare findings and resolve any discrepancies. Full texts were obtained and screened again by both reviewers. The two reviewers met again and resolved discrepancies without needing to consult a third reviewer. Key journals were hand searched, as well as reference lists of key papers.

Box 1 lists the inclusion and exclusion criteria.

**Box 1.** Search criteria.

Inclusion criteria
• Empirical studies published in English
• Studies involving people with dementia or cognitive impairment, or relatives and friends of people with dementia or cognitive impairment
• Studies employing recognised qualitative methods, including mixed methods studies
• Studies examining the process of help seeking in relation to the diagnosis of dementia
Exclusion criteria
• Studies which only include the views and experiences of healthcare professionals
• Studies of the help-seeking behaviour of first-degree relatives of people with dementia who are worried about their own cognitive health (e.g. because of genetic factors)
• Studies describing help seeking *post* diagnosis

### Quality assessment

**Figure 1.**
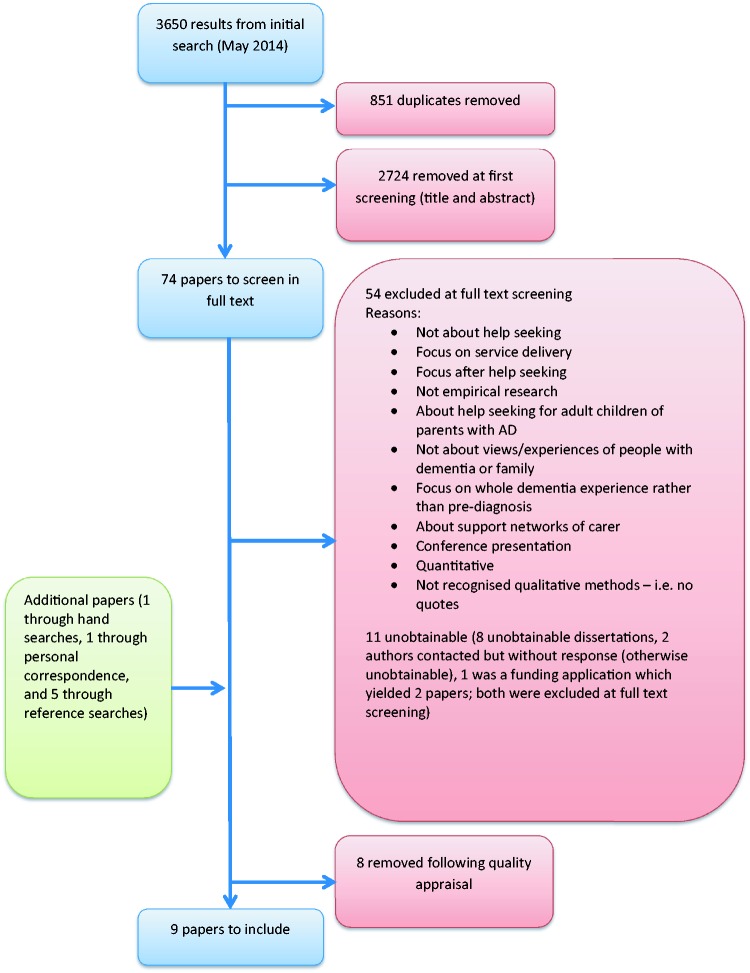
Flow chart showing selection of studies.

Although the practice of appraising the quality of qualitative research for use in reviews and syntheses is often debated (Campbell et al., 2011; Hammersley, 2002; Popay, Rogers, & Williams, 1998), our initial readings indicated that some of the papers were of questionable rigour and so we decided that quality appraisal was necessary. Two reviewers (LP and GO) independently assessed the 16 studies using the Critical Appraisal Skills Programme (CASP) assessment tool for qualitative research (CASP, 2013). A further eight papers were excluded because of issues with rigour and, in some cases, because only a very small section of the paper was relevant to the pre-diagnosis period (for example, Brown & Chen, 2008 and Chrisp, Tabberer, Thomas, & Goddard, 2012, respectively). Figure 1 illustrates how the final selection of papers was made.

### Data extraction

A data extraction form was used to extract the main concepts, sub-concepts and participant quotes from each paper; this forms the data for the synthesis.

Table 1 describes the basic study information of each paper.

**Table 1.** Basic study information for each included paper.

Author/s	Year	Journal	Country	Aim of the study	Dementia type	Participants	Cultural group	Data collection	Data analysis
Robinson et al.	1997	*Health Care in Later Life*	Sweden	To describe the experience of early memory loss and the transition towards seeking professional help	‘Early symptoms of memory loss’	People with dementia	Not stated	Semi-structured interviews	Thematic
Keady	1999	Thesis	UK (Wales)	To explore the temporal process of coping within dementia, grounding any emerging insights in the experience of family caregivers themselves	‘Dementia’	Carers^a^	Not stated	Semi-structured interviews	Constant comparative method (Grounded Theory)
Brown and Alligood	2004	*Journal of Applied Gerontology*	USA	To explore patterns of help-seeking by older wife caregivers of husbands with dementia	Any	Carers (wives)	Caucasian	Unstructured interviews	Constant comparative method (Grounded Theory)
Krull	2005	*Journal of Aging Studies*	USA	To shed light on the process through which familial caregivers decide to seek out a formal diagnosis of Alzheimer’s disease in their loved ones	AD	Carers	White	Semi-structured interviews	Constant comparative method (Grounded Theory)

Leung et al.	2011	*Health and Social Care in the Community*	Canada	To explore the perceptions and experiences of problem recognition, and the process of obtaining a diagnosis among individuals with early stage dementia and their primary carers	Any	Both	Anglo-Canadian	Semi-structured interviews	Qualitative descriptive approach (Sandelowski, 2000)
Mukadam et al.	2011	*International Psychogeriatrics*	UK	To explore the effect of culture and ethnicity on beliefs and attitudes of carers to help-seeking for dementia symptoms and the impact of these beliefs on the pathway to diagnostic and therapeutic services	Any	Carers	Seven different ethnic groups	Semi-structured interviews	Thematic
Van Vliet et al.	2011	*International Psychogeriatrics*	Netherlands	To investigate the barriers to obtaining a dementia diagnosis for caregivers of people with EOD and to develop a typology of the diagnosis pathway	EOD	Carers	Not stated	Semi-structured interviews	Constant comparative method (Grounded Theory)

Koehn et al.	2012	*Journal of Aging Studies*	Canada	To understand the experiences of our participants as they negotiate the pathway to a diagnosis of dementia for themselves or a family member	ADRD	Both	Chinese-Canadian	Semi-structured interviews	Thematic
McCleary et al.	2013	*Dementia*	Canada	To explore and describe the experiences of South Asian Canadian persons with dementia and their family carers in the time prior to a diagnosis	Any	Both	South Asian Canadian	Semi-structured interviews	Qualitative description and content analysis

ADRD: Alzheimer’s disease and related disorders/dementias, AD: Alzheimer’s disease, EOD: early onset dementia.

a Keady developed two separate models from his PhD work: a six-stage model based on carer interviews, and a nine-stage model based on data from people with dementia. We decided to include the six-stage model because it was based on a much greater amount of data.

### Synthesis: The meta-ethnographic approach

Noblit and Hare (1988) developed the meta-ethnographic method of synthesizing qualitative research, in which the aim is to create translations, rather than generalisations. In a meta-ethnography, each study is reduced to its key concepts, and the concepts of each study are translated into one another in an effort to establish similarities and differences between them. According to Noblit and Hare, “The translation of studies takes the form of an analogy between and/or among the studies.” (1988, p. 10). The meta-ethnographer ‘translates’ by taking each key concept from the first paper, comparing it closely with similar concepts in the second and subsequent papers, and drawing analogies of the type ‘X is like Y except for Z’.

Noblit and Hare describe three ways in which studies are related to one another:

- The accounts are directly comparable and their translations do not reveal opposition, therefore they are ‘reciprocal’ translations.
- The accounts stand in opposition; these are ‘refutational’ translations.
- The studies taken together represent a ‘line of argument’.

Britten et al. (2002) describe three levels of thinking that are brought together in a meta-ethnography: ‘first-order’ concepts or interpretations of the phenomenon being studied (those of the primary research participants, represented by original quotations); ‘second-order’ concepts or interpretations (those of the primary researchers); and ‘third-order’ concepts or interpretations (those of the reviewers or secondary researchers). First and second order concepts are extracted from each of the primary studies. Third-order concepts are developed during the synthesis stage.

Following data extraction, we populated a table with the second-order concepts from each paper, in order to assess the degree of reciprocity between them. After juxtaposing them in this way, we then translated the concepts of the papers into one another. We started by comparing just two of the studies. Once these two studies’ concepts had been closely compared, the list of translations that was produced was compared with the next study’s concepts. This process continued until all the papers had been translated into one another. The resulting list of concepts was then taken back to the original studies in a fashion not dissimilar to the constant comparison method used in Grounded Theory (Glaser & Strauss, 1967). This enabled us to make inferences about what the studies told us when taken together as a whole and thus develop a line-of-argument synthesis.

## Findings

The views of 249 participants were represented, including 32 people with dementia and 217 carers (171 spouses, 39 children, six sons- or daughters-in-law and one sibling). Of the carers, the majority were female (60%), while the majority of people with dementia were male (59%). Three of the studies were conducted in Canada, two in the USA, two in the UK, one in the Netherlands and one in Sweden. All the studies employed unstructured or semi-structured interviews but different approaches and methods of analysis were described (see Table 1).

**Figure 2.**
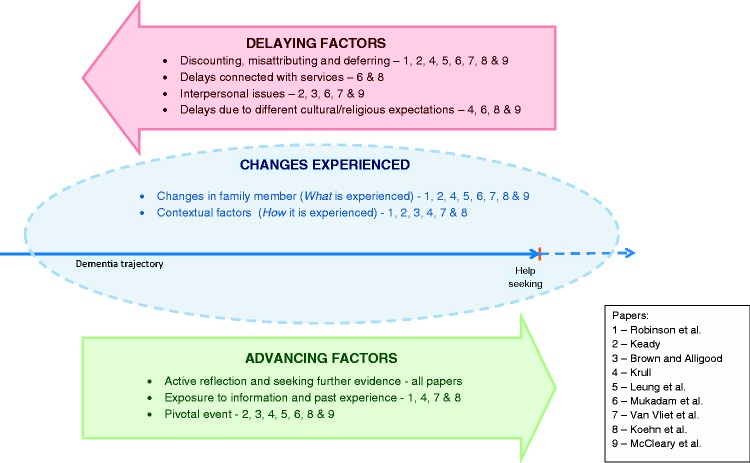
Summary of findings showing contributions of each paper to concepts.

The main concepts were ordered under three groups: changes experienced, delaying factors and advancing factors. Figure 2 shows which papers contributed to each concept. Where quotes are included, speech marks denote study participants’ words (i.e. first-order interpretations) and inverted commas denote primary researchers’ words (second-order interpretations).

### (i) Changes experienced

This concept was evident in all of the papers, though the authors used different words for it, such as changes in the family member and disrupted family life (Van Vliet et al., 2011), awareness of early signs (Leung et al., 2011) and first signs and contrary behaviour (Krull, 2005). The majority of papers spoke about changes in terms of both *what* was experienced (changes in the family member) and *how* it was experienced (contextual factors).

#### Changes in the family member

Changes in the family member included memory and cognitive changes, such as becoming more forgetful, not being able to find the right word and a general loss of ability to perform usual tasks. There were also instances of changes in behaviour, personality and relationships. Leung et al. (2011) reported that, in people with dementia, “behaviours became increasingly inconsistent with how they ‘used to be’.” Other participants noted a general loss of interest in things and a change in the relationship between the person with dementia and carer:

“The behaviour of my husband changed. He just did not respond when I needed help or when I needed an arm around my shoulder.” (Van Vliet et al., 2011)

Sometimes this change in the relationship was more dramatic:

“I just got me a bite to eat … I was sitting there and he came and he said, “are you sleeping?” And I started to say, “no,” and he said, “Well, this will wake you up. In the morning, I’m calling a lawyer, I want a divorce!” (Brown & Alligood, 2004)

Koehn et al. (2012) and Van Vliet et al. (2011) both noted that only carers mentioned the personality and relationship changes. Studies that interviewed both people with dementia *and* carers found that only carers noticed personality and relationship changes; those that interviewed only carers mostly^1^ reported both memory changes and personality/relationship changes, and the one study that interviewed only people with dementia only reported memory changes. The types of change reported by people with dementia and carers in each study are shown in Table 2.

**Table 2.** Types of changes reported by the person with dementia and carers.

	Robinson et al.	Keady	Brown and Alligood	Krull	Leung et al.	Mukadam et al.	Van Vliet et al.	Koehn et al.	McCleary et al.
Participants	Person with dementia	Carers	Carers	Carers	Person with dementia and carers	Carers	Carers	Person with dementia and carers	Person with dementia and Carers
Changes reported	Memory	Memory & behavioural/personality	None reported	Memory/ cognitive & behavioural/ personality	Memory & behavioural/personality	Memory (first)	Memory & behavioural/personality	Memory & behavioural/personality	Memory & behavioural/personality
Additional					Person with dementia noticed memory/ cognitive while carers noticed behavioural/ personality	Note: only one sentence on changes noticed		Person with dementia noticed memory/ cognitive while carers noticed behavioural/ personality	Authors say that person with dementia gave fewer details than carers about early signs

#### Contextual factors

Most of the papers discussed the contextual factors that influenced how the changes were experienced. Some highlighted the social manifestations of the changes, such as their impact on work and family life (Brown & Alligood, 2004; Krull, 2005; Leung et al., 2011; Van Vliet et al., 2011), while others commented on the variable rates of decline (Koehn et al., 2012; Leung et al., 2011). Social manifestations included family and marital conflict and tensions in the work place:

“It was a terrible period in which you would rather stay away from home because of the tension my husband brought. He was very nasty to the children.” (Van Vliet et al., 2011)

“She experienced a lot of grief; she did not understand anything about all the accusations she got at work. They accused her of neglecting things at work and I had to go out of my way to comfort her.” (Van Vliet et al., 2011)

Four papers noted that the realisation that the events or behaviours were deviant or indicative of dementia occurred in retrospect. Keady (1999) adds to this concept the idea of noticing as an active process rather than a single moment in time.

### (ii) Delaying factors

Delaying factors contained four distinct sub-concepts:

- Discounting, misattributing and deferring;
- Interpersonal issues;
- Delays due to different cultural expectations;
- Delays due to services.

#### Discounting, misattributing and deferring

With the exception of Brown and Alligood (2004), all papers made at least some reference to the discounting and misattribution of symptoms. A behaviour can be discounted by being regarded as normal for that person (consistent with their usual self), normal compared with other people, or part of normal ageing:

*Normal for the person:* “My wife always was a forgetful person, so these problems were not obvious for me.” (Van Vliet et al., 2011)

*Normal compared with others:* “We all forget things” (Leung et al., 2011)

*Normal aging:* “I knew something was not right. I thought well, it’s just her age” (Krull, 2005)

#### 

As Keady (1999) points out, even when events are normalised in this way, they may not be fully discounted and will influence the interpretation of future events.

Behaviours and events can also be misattributed; they are thought to be caused by something other than dementia, such as another physical illness, stress, or trauma:

“Because of his illnesses, his diabetes and … an open bypass heart surgery … they thought maybe his illness causes him [memory problems].” (Mukadam, Cooper, Basit, & Livingston, 2011)

“Our mother died last year and we all put it down to him being sad and depressed about it all”. (Keady, 1999)

In one paper, Van Vliet et al. (2011), the authors include ‘noticing a problem but not attributing to anything’ as a misattribution. We consider, however, that noticing a problem but not attributing it to anything is neither discounting nor misattributing, as the following quote demonstrates:

“If your wife always has been active, but stops doing that all of a sudden, you think: ‘what is going on?’ However, I never knew what to make of it; I was never able to label this behaviour. I only thought it was very annoying.” (Van Vliet et al., 2011)

McCleary et al. (2013) also talk about carers’ ambivalence about what the cause of the changes might be. We would interpret both this ambivalence and ‘noticing a problem but not attributing to anything’ (Van Vliet et al., 2011) as ‘deferring’; a decision about the cause of the event is simply deferred or put to one side until the person is better able to make sense of and evaluate it. We make the distinction then between when the person thinks the behaviour or event is normal (discounting), when they think it is due to another cause (misattributing), or when they are unsure what the cause is and thus reserve judgement for the time being (deferring).

#### Interpersonal factors

Only four of the papers contributed to this concept (Brown & Alligood, 2004; Keady, 1999; Mukadam et al., 2011; Van Vliet et al., 2011). In all cases, some level of denial was reported, either on the part of the person with dementia (Keady, 1999; Mukadam et al., 2011; Van Vliet et al., 2011), who denied the problem or tried to hide the signs, or on the part of the carer (Brown & Alligood, 2004), who minimised the problems as a means of coping. Denial of a problem, on part of the person with dementia or a family member, makes communication about the changes difficult.

Carer: “And I kept telling myself, “Well, it’s not Alzheimer’s. They don’t know what they’re talking about because this man is perfectly normal!” When all the time I, I knew, but just did not want to accept it.” (Brown & Alligood, 2004)

It is not clear in either case whether the act of minimising or denying was intentional. It is particularly unclear in the case of the person with dementia because lack of awareness is a manifestation of the illness.

When people with dementia cover up the signs that something is wrong, carers can gain little understanding and confirmation from their social network members, who may not notice the ‘covered-up’ problems (Van Vliet et al., 2011):

“That period was very tiring for me because I could not convince anyone that something was wrong. My husband …, knew very well how to manage in specific situations, so nobody noticed anything. He could really fool people.” (Van Vliet et al., 2011)

Thus, when the person with dementia denies changes and hides the signs, this can cause interpersonal strains between them and the caregiver and delay help-seeking. This can lead to a moral dilemma for the carer, who may have noticed a problem but is reluctant to seek help because they feel they would be betraying the person with dementia or breaching their confidence (Keady, 1999; Mukadam et al., 2011):

“He [carer] didn’t want to say anything because he was too frightened in case he lost her [the person with dementia’s] trust.” (Mukadam et al., 2011)

This moral dilemma can be heightened by respect for the family hierarchy, and a feeling of a family duty to care. The carer may also wish to protect the person with dementia, particularly when they have concerns about the stigma of mental illness (Mukadam et al., 2011).

#### Delays due to different cultural or religious expectations

Four of the studies made contributions to this concept (Koehn et al., 2012; Krull, 2005; Mukadam et al., 2011). Two of these (Koehn et al., 2012; Krull, 2005) are directly and reciprocally translatable, except that they apply to two different cultures: American and Chinese. The argument put forward in both papers is that there is a cultural or religious expectation that forgetfulness is a normal sign of aging. Given that the majority of the other papers also make reference to participants’ beliefs that the changes they were experiencing were a normal part of aging, it could be assumed that this is a much wider cultural phenomenon, and not specific to these two cultures.

Another study to contribute to this concept, Mukadam et al. (2011), gives an example from an Asian culture, where there was an expectation that, when a son marries, his wife takes over the household tasks and so the parent’s loss of ability to perform those tasks may be less noticeable. McCleary et al. (2013) describe how one of their participants viewed the changes as natural in connection with a Hindu deity, Lord Shiva:

“I think, well first of all this is natural. You can’t help too much. This is, everything is, on a decline … this Law of Shiva is working. Everything is deteriorating, getting back to the recycle process.”

#### Delays due to services

In Koehn et al.’s (2012) study, new immigrant caregivers faced particular difficulties in accessing resources:

“I did not know [about support services] because although I was here for nearly three years, I was at home [caring for my child and mother]. I don't know many people here. I did not come across these social services and their information before … Moreover, I do not know what type of social services are available to these patients in Canada.”

In some cases, a high level of trust in the services resulted in delayed help seeking, as it was expected that the doctor would pick up on the changes:

“No the doctor they keep seeing him they don’t tell me anything … I rely on the GP.” (Mukadam et al., 2011)

In other cases the opposite was true, and the carer delayed seeking help because of a lack of trust in the GP or in psychiatry services (Mukadam et al., 2011).

### (iii) Advancing factors

Mukadam et al. (2011) term this concept ‘factors precipitating help seeking’. The term was changed to ‘advancing factors’ as this encompasses more concepts that refer to factors which facilitate acknowledging, discussing and observing as well as seeking medical help, and thus advancing progress along the trajectory towards diagnosis. This main concept has the following sub-concepts:

- Active reflection and seeking further evidence;
- Exposure to information and past experience;
- Pivotal event.

#### Active reflection and seeking further evidence

Some participants described processes of active reflection and the seeking of further evidence. For some carers this meant ‘increased surveillance’ of the person with dementia and ‘checking out’ with family members (Keady, 1999). In some cases, information was actively sought from family and social network members (Keady, 1999; Robinson, Ekman, Meleis, Winblad, & Wahlund, 1997), while in other cases the network members were forthcoming with their observations (Brown & Alligood, 2004; Krull, 2005). In either case, the observations of others were compared with the participant’s own observations and were used as a basis for re-assessment of the situation:

“His brother also talks to me and then sometimes when he says something I took it lightly. He says ‘No. No. There’s nothing to take, it’s not a laughing matter. We have to look into it. It can be serious. He needs some treatment, attention.’ So I told him, ‘Let’s go to the doctor and see.’” (McCleary et al., 2013)

The accumulation of events also prompted comparison and reflection and led to a process of ‘gradually piecing together’ (Brown & Alligood, 2004; Koehn et al., 2012; Leung et al., 2011). This process of active reflection and seeking of further evidence leads to the redefinition of the situation. The new definition of the situation can then be used as a benchmark against which to measure further events, or it may be sufficient in itself for the participant to want to seek medical help.

The people with dementia in the study by Robinson et al. (1997) described a similar process of active reflection. They reported a gradual awareness that the changes they experienced were no longer random or isolated experiences in their lives, so that they were no longer able to normalise them.

“I had been shopping and forgot my groceries in the store … I didn’t even realize as I came home or when I got to the car, and then I actually started wondering about myself”

#### Exposure to information and past experience

Of the four papers that contribute to this concept, three mentioned recognition of dementia on the basis of previous family experience of the disease (Krull, 2005; Robinson et al., 1997; Van Vliet et al., 2011), while the fourth introduced the idea of increased recognition of dementia on the basis of professional knowledge of the disease, or exposure to information about it (Koehn et al., 2012). When engaged in active reflection, the participant can draw upon, and make comparisons with, their knowledge stock and their experiences of other people’s dementia. Again, this can contribute to a redefinition of the situation and advancement along the trajectory to diagnosis.

#### Pivotal event

At times, the period of active reflection and seeking further evidence was cut short due to a pivotal event that caused dramatic recognition of the need to seek medical help. Most commonly this took the form of a crisis or potentially dangerous event that caused participants to recognise that the situation was more serious than they thought (Brown & Alligood, 2004; Keady, 1999; Koehn et al., 2012; Krull, 2005; McCleary et al., 2013). Examples included traffic accidents, getting lost and burning saucepans. In other cases, the pivotal event consisted in participants being told by their doctor that they had dementia when they had consulted the doctor about another problem (Koehn et al., 2012; Mukadam et al., 2011). A unique example of a pivotal event is described by Leung et al. (2011), where a person with dementia spontaneously disclosed their memory problems while visiting the doctor for another reason:

“It was a flip comment when I was walking out of the doctor’s office … I said, ‘Oh by the way can you do anything for memory loss?’”

In this latter example, the snap judgement of the participant to disclose their concerns to the doctor was pivotal in the advancement of the trajectory towards a diagnosis.

## Discussion

### Line of argument synthesis

Table 3 shows the key concepts from the papers, the primary authors’ second-order interpretations and our third-order interpretations.

**Table 3.** Key concepts and second- and third-order interpretations.

Concepts		Second-order interpretations	Third-order interpretations
Changes experienced	Changes in family member	- Changes include memory, cognition, loss of abilities, behaviour and personality	People with dementia do not notice, or at least do not admit to, the personality/relationship changes^a^
	Contextual factors	- Noticing as process and realisation through retrospect - Changes manifest in family relationships and work contexts - Rate of decline affects how changes are perceived	- Social manifestations such as relationship and work tensions sometimes act as the sign that something is not right rather than the symptoms themselves - When a new situation (sign, event, change, etc.) occurs, it is evaluated by the person who notices in the context of changes they may have previously noticed. In this way, past events help to shape the evaluation of the current event, and, at the same time, past events are re-evaluated in light of the new events; thus some signs are only ‘noticed’ in retrospect.
Delaying factors	Discounting, misattributing and deferring	- Attributed to: Normal aging, concurrent health issues, emotional trauma or stress, person with dementia feigning the problem, malicious and intentional behaviour, or noticed change but did not attribute to anything - Although normalised, the event is not discounted completely and will influence the interpretation of future events - Ambivalence exists about the cause of the changes	There are three possible responses to the signs of dementia: 1 – Discounting – as either normal aging (‘that happens when you get older’), normal as compared to others (‘we all do that’), or normal compared to the person with dementia’s usual self (‘She’s always been like that’) 2 – Deferring – no immediate judgement is made and the information is ‘shelved’ until the person can evaluate it 3 – Misattributing – there is thought to be another cause for the changes, for example high blood pressure or stress at work
	Interpersonal issues	- Person with dementia’s denial and refusal to seek help - Person with dementia’s covering up leads to lack of understanding and confirmation from the social context - Carers worried about upsetting or betraying the person with dementia - Caregivers can minimise the problems as a means of coping - Family duty to care – therefore help not needed - Respect family hierarchy - Concerns about the stigma of mental illness	The person with dementia’s reluctance to accept a problem and seek help causes a moral dilemma for the significant other who may feel that seeking medical help or consulting lay networks would be a betrayal of the person with dementia’s confidence.
	Delays due to different cultural explanations	- Aging is considered normal in the context of American culture and Chinese culture - Cultural expectations can influence whether or not a problem is recognised – e.g. the expectation that a woman’s household duties cede to her daughter in law. Therefore the loss of abilities was not picked up. - attributed to religious causes	Different cultural norms and values influence the ways in which experiences are defined (e.g. as a normal part of aging), as well as the expected responses to these experiences (e.g. the family duty to care).
	Delays connected with the services	- Delays because doctor is trusted to identify the problem - Lack of trust or information in the health care system - Importance of system and resource accessibility	
Advancing factors	Active reflection and seeking further evidence	- Symptom appraisal – development of hypotheses about the cause of the observed symptoms - Observations of others - Increased surveillance - Person with dementia’s strategies gradually failing, they become more reflective about their actions and abilities and compare to themselves and others - Reflecting on future needs - Accumulation of problems leads to recognition - No longer able to normalise	People with dementia and their families try to make sense of their experiences by observing, comparing and reflecting. As new experiences occur, the whole situation is redefined and new hypotheses developed about the cause of the changes. As problems accumulate, as long as they are reflected upon, the closer the hypothesis will be to dementia as the cause
	Exposure to information or past experience	- Past experience of dementia with another family member - Professional knowledge - Exposure to information about dementia	Past experience and knowledge of dementia add to the resources with which people make sense of what is happening
	Pivotal event	- Something happens that forces them to acknowledge a problem. It can no longer be discounted, misattributed or deferred. This is usually a crisis or safety concern. - Dementia recognised when seeking help for another problem - Carer’s patience finally snaps and they confront the person with dementia	A pivotal event causes the recognition of a need to seek medical help, or at least a sudden advancement along the trajectory to diagnosis At the time of this recognition the person may not have hypothesised dementia, or they may have hypothesised it some time ago

a Koehn et al. introduced this concept but the other papers are in support of it when taken together – see Table 2.

By translating the concepts and synthesising these translations we are able to propose the following line of argument. The early signs of dementia are experienced not only as changes in memory and cognitive functioning, but also as changes in personality and strains in interpersonal relationships within family and work contexts. It seems that people in the early stages of dementia tend to be more aware of the memory and cognitive changes and not the changes in their personality and relationships. This contributes to the moral dilemma faced by family members, who may have noticed changes but, due to lack of awareness or denial on the part of the person with dementia, may feel unable to do anything about it through fear of upsetting or betraying the person. Noticing occurs as a process, in which new events are evaluated in relation to the stock of knowledge the individual holds, which is influenced by their level of medical understanding and past experiences.

When an event occurs, individuals respond by either generating a hypothesis about the cause of the problem, or simply deferring judgement about the cause of the event until a later date. When hypotheses are generated, they contribute to the stock of knowledge held by the individual that is used to interpret future events; past events help to shape the evaluation of the current event and, at the same time, past events are re-evaluated in light of new events. Freeman’s (2010) work on narrative reflection has relevance here; Freeman states that ‘self-understanding occurs in significant part through narrative reflection, which is itself a product of hindsight’*.* He asserts that through hindsight and narrative reflection, things can be seen that could not have been seen at the time the event occurred.

The accumulation of events causes suspicion and individuals begin to actively monitor the situation, gather information from others, and compare and reflect. Pivotal events can occur which cause a rapid advancement along the trajectory towards a diagnosis, either through sudden recognition of a problem, sudden recognition of dementia, or sudden recognition of the need to seek medical help. These pivotal events are usually crises that give rise to safety concerns or great tensions in relationships.

The following metaphor helps to express this line of argument. Consider each unusual event as an object that a person comes across and needs to deal with. They must make an assumption about what the object is and ‘file’ it accordingly in a cupboard. They might assume that this unusual event is normal behaviour, or the result of another health problem. If the person is unsure about what the object is (what the event means), they might file it provisionally before looking for similar objects to confirm or disprove their suspicions (surveillance and seeking the opinions of others). If a person is not ready to make a judgement about what the object is or cannot face thinking about it at that time, they may just throw it in the cupboard without worrying about where it lands, and close the door. In this case no assumptions have been made about the object but it is still accessible in the cupboard. As the individual comes across further objects, the same process is followed, but this time there is a further option: to look at the objects already in the cupboard, compare them with the new object and use them to decide where to place it. In this way, as the person views what is already in the cupboard, they might also decide to reorganise the contents according to new assumptions. If objects in the cupboard are allowed to simply accumulate without the person ever taking stock of where the objects relate to each other, there is a risk of the cupboard reaching full capacity and bursting open. This represents a pivotal event, where stress and strain may have been mounting for some time and the door bursts open and the contents (and their underlying meanings) are clear to see.

These findings appear to support much of the evidence from the help-seeking literature. As the literature suggests, the experiences of individuals that are represented and interpreted within these nine studies show that help seeking is a complex process of recognising changes and making sense of them within the individuals’ existing spheres of knowledge. This review also demonstrates a clear reliance on lay networks in making attributions about the cause of changes, as is described by Cornford and Cornford (1999), Freidson (1970) and Kleinman (1980). While the ‘dynamic’ models of help seeking (Biddle et al., 2007; Dingwall, 1976; Leventhal et al., 1984; Pescosolido, 1991, 2006; Saint Arnault, 2009; Wyke et al., 2013) account for the influence of social networks on decision making, they do not specifically account for those situations in which a family member or friend has to interpret the changes and act on behalf of the ill person. In a study about the role of family members and friends in recognising and responding to mental distress, Owens et al. (2011) draw attention to the moral dilemma faced by those who must act on behalf of a significant other. They may recognise the need both to alert other members of the social network and to seek professional help, but fear that doing so would constitute betrayal and might damage their relationship with the distressed person, thus cutting off a vital source of social support. This review reports similar experiences reported by family members, who felt that discussing changes with others and seeking professional help would breach the trust of the person living with dementia.

### Limitations of existing literature and implications for further research

Although this synthesis provides a strong line of argument about the ways in which people weigh up the meaning of individual experiences, several concepts (delays due to people with dementia, delays due to families and delays due to cultural expectations) suggest that there is a strong interpersonal dimension to the way in which people make help-seeking decisions. While we have been able to account for these concepts and create third-order interpretations about them, information in the individual studies about these issues was sparse and underdeveloped. Thus the line of argument that emerges from existing studies does not sufficiently reflect the whole experience of recognising and responding to the signs of dementia.

Further work is needed to draw out some of the complexities involved in the interpersonal aspects of decision making, including the effects of familial and cultural practices and the moral dilemmas faced by family members who are worried about upsetting or betraying the person with dementia. The review has highlighted that looking back on events through hindsight, and weighing and corroborating them, enables people to make sense of individual experiences in the light of previous experiences. Family members may be concerned that engaging in such reflective practices, including actively gathering evidence and weighing it up, may be a serious breach of the person’s autonomy and thus risks upsetting or alienating them.

Future work might also address the following areas:

- Which signs and symptoms people with dementia can and cannot become aware of, and how this affects communication with and between family members?
- How commonly people notice personality changes and tensions in relationships and what they attribute these to?
- How people utilise their lay referral networks, including which network members they seek advice from?
- The moral dilemmas faced by family members about upsetting or betraying the person they are concerned about.

### Implications for practice

A number of implications for practice also arise from the findings of this review, mainly in the area of public health. Current public health messages about early signs of dementia focus heavily on memory changes and loss of cognitive abilities. This review highlights other important early signs, such as behavioural and personality changes, and the social manifestations of dementia in familial and work relationships. More recent research has drawn attention to changes in sense of humour (Clark et al., 2015). Increasing public awareness of these more subtle and diverse changes might enable people to make earlier and more accurate hypotheses about problems they are experiencing and their possible causes. A development of the metaphor we described could encourage people to sort the objects as they are placed in the ‘cupboard’, instead of just throwing them in, and to review the contents regularly.

As the findings have shown, experiences are often allowed to accumulate before a person decides to review and evaluate them in light of each other. A public health message could encourage people to weigh up and compare their experiences both with those of other people and with their own experiences from previous years. This reflection allows individuals to consistently evaluate problems and make and test hypotheses. The trajectory toward a diagnosis is then more likely to be characterised by a smooth and steady growth of realisation, rather than a relative lack of awareness until a pivotal event occurs. For the person with dementia, this could mean that they receive the diagnosis while they still have chance to make sense of it and plan for the future, and for some it could also mean the earlier commencement of medications that can potentially slow the decline.

A final implication for practice stems from the concept of ‘delays due to services’. Both Koehn et al. (2012) and Mukadam et al. (2011) reported instances of delays because doctors had not recognised the signs of dementia, delays because of lack of trust in services, or delays due to lack of knowledge of the system among recent immigrants. This suggests that more could be done to improve clinical decision making and to engage minority groups who may face specific barriers to accessing services.

## Conclusion

Systematic searching, screening and appraisal allowed us to identify nine high-quality qualitative studies of the ways in which people come to recognise and respond to the early signs of dementia. Data were extracted from each paper and, following a meta-ethnographic approach, these were translated into one another to produce key concepts, namely: the changes that were experienced; factors that delayed help seeking, and factors that advanced the help-seeking process. Taken together, the papers present a common line of argument.

The argument suggests that events are not experienced in a social and historical vacuum and that the recognition of dementia and the need to seek medical help is part of a long process, rather than a single moment in time. Individuals notice a wider range of signs than just memory and cognitive changes; personality changes and general tensions in relationships at work and at home are also reported. Active reflection on the meaning of events and gathering of further evidence appears to lead to earlier recognition of the need to seek medical help; it also reduces the risk of a pivotal event that could potentially be a crisis situation. This process occurs as a repeating cycle of noticing, attributing, developing hypotheses and observing, comparing and reflecting. Familial and cultural factors can serve to delay medical help seeking, including fear of upsetting the person with dementia and feeling a duty to care for them within the lay network, but further work is needed in this area to understand these interpersonal issues and their impact.

## Authors’ note

The views expressed are those of the authors and not necessarily those of the NHS, the NIHR or the Department of Health.

## Note

1 The exception to this is in Mukadam et al. who only ascribe one sentence to the changes noticed: ‘nearly all the carers reported that the first symptom they noticed was forgetfulness’. Therefore, other symptoms may also have been noticed after the forgetfulness, but this is not reported. Because only one sentence is afforded to this, and because the authors say that forgetfulness is the *first* (not necessarily the *only*) symptom, this was not considered as a refutation.

## Appendix

### Search strategy as run in Ovid platform

*Database*: Ovid Medline(R) In-process and other non-indexed citations and Ovid Medline(R) 1946 – present; Embase 1974 to 2015 May; PsycINFO 1967 to May 2015

*Host*: Ovid

*Strategy*:

1. exp Dementia/
2. dementia.ti,ab.
3. alzheimer*.ti,ab.
4. cognition disorders/ or mild cognitive impairment/
5. cognition.ti,ab.
6. (cognitive adj (impairment* or disorder* or disease*)).ti,ab.
7. (memory adj2 loss).ti,ab.
8. 1 or 2 or 3 or 4 or 5 or 6 or 7
9. family.ti,ab.
10. husband.ti,ab.
11. spouse.ti,ab.
12. wife.ti,ab.
13. Children.ti,ab.
14. carer.ti,ab.
15. partner*.ti,ab.
16. (social adj network*).ti,ab.
17. exp Social Environment/
18. (significant adj other*).ti,ab.
19. (care adj giver*).ti,ab.
20. caregiver*.ti,ab.
21. 9 or 10 or 11 or 12 or 13 or 14 or 15 or 16 or 17 or 18 or 19 or 20
22. Early Diagnosis/
23. (early adj (diagnos*or detection or identif* or recogni*)).ti,ab.
24. (timely adj (recogni* or detection or identif* or diagnos*)).ti,ab.
25. (help adj seek*).ti,ab.
26. pathway.ti,ab.
27. exp Sick Role/
28. exp Illness Behavior/
29. trajectory.ti,ab.
30. (illness adj behaviour).ti,ab.
31. (illness adj behavior).ti,ab.
32. 22 or 23 or 24 or 25 or 26 or 27 or 28 or 29 or 30 or 31
33. 8 and 21 and 32
